# *PDE4D* single nucleotide polymorphism rs918592 is associated with ischemic Stroke risk in Chinese populations: a meta-analysis

**DOI:** 10.1186/s12872-023-03681-2

**Published:** 2024-01-03

**Authors:** Xinrui Yu, Guiying Zhang, Xuelei Tang, Rong Lin

**Affiliations:** 1 Department of Biology, Hainan Medical University, Haikou, China; 2 Center of Forensic Medicine of Hainan Medical University, Hainan Provincial Academician Workstation (tropical forensic medicine), Hainan Provincial Tropical Forensic Engineering Research Center, Haikou, China

**Keywords:** PDE4D, Rs918592, Single nucleotide polymorphism, Ischemic Stroke, Meta-analysis

## Abstract

**Background:**

Several studies have investigated the correlation between phosphodiesterase 4D (*PDE4D*) single nucleotide polymorphism (SNP) rs918592 and the risk of ischemic stroke (IS) in Chinese populations. But the results were inconsistent and inconclusive. Therefore, to resolve this conflict, we conducted a meta-analysis to further elucidate their relationship in Chinese populations.

**Methods:**

Studies focused on SNP rs918592 and IS risk were electronic searched in the databases of PubMed, Embase, ISI Web of Science, Weipu, China National Knowledge Infrastructure (CNKI), Chinese Biomedical (CBM) and Wanfang. The association between SNP rs918592 and IS risk was expressed by odds ratio (OR) with its confidence interval (CI). Begg’s and Egger’s linear regression tests were used to assess publication bias. The meta-analysis was performed with STATA 11.0 statistical software. Two online prediction websites (HaploReg and RegulomeDB) were adopted to explore the functions of SNP rs918592.

**Results:**

The meta-analysis ultimately included 10 studies involving 2,348 cases and 2,289 controls. The results showed that there was a significant correlation between SNP rs918592 and IS risk in Chinese individuals. The G allele had reduced risk of developing IS compared to the A allele (OR 0.83, 95% CI 0.74–0.95, *P* = 0.005). HaploReg and RegulomeDB analyses suggested that SNP rs918592 and its strongly linked SNPs (e.g. rs34168777) might have regulatory functions.

**Conclusion:**

This study shows that SNP rs918592 in *PDE4D* may be a contributor of IS risk in Chinese populations. It offers a good answer for the association of *PDE4D* SNP rs918592 with IS risk in Chinese populations for the first time.

**Supplementary Information:**

The online version contains supplementary material available at 10.1186/s12872-023-03681-2.

## Introduction

Stroke is one of the most frequent contributors to disability and mortality in the world, including China. In China, according to a cross-sectional survey of stroke burden in 155 urban and rural centers in 31 provinces, the crude stroke incidence rate was 345.1/100,000 person-years [1]. Ischemic stroke (IS) is the most common type of stroke, which makes up approximately 80% of all stroke cases. The occurrence and development of most IS is the result of the interaction between genetic and environmental risk factors.

Phosphodiesterase 4D (PDE4D), which can specifically degrade cyclic adenosine monophosphate (cAMP), has been implicated in the pathogeny of IS. It is expressed in many cells such as immune cells (T lymphocytes, macrophages, and monocytes), endothelial cells, smooth muscle cells, and atrial myocytes [2–4]. A decrease in the cAMP concentration promotes vascular smooth muscle proliferation [5]. Conversely, an increase in cAMP attenuates the formation of neointima and suppresses vascular smooth muscle proliferation after arterial injury [6]. PDE4, including PDE4D, are the major enzymes of cAMP signal transduction pathway in inflammatory cells. A decline in cAMP leads to inflammation [7]. Inflammation may contribute to atrial fibrillation and atherosclerosis, both of which are risk factors for IS.

In 2002 and 2003, the deCODE Genetics group conducted linkage and association analyses in the Icelandic population and identified *PDE4D* as a susceptible gene of IS [8, 9]. Since then, whether the genetic variants of this gene are related to stroke has become a research hotspot. In Chinese populations, single nucleotide polymorphism (SNP) rs918592, lying in an intron near the 5’ end of *PDE4D*, has been investigated whether it was associated with IS risk. But the results of different studies were discordant. For instance, Tang (2007) [10] reported a negative result, while Xu et al. (2008) [11] and He et al. (2012) [12] reported positive results. Therefore, in order to further elucidate the correlation between SNP rs918592 and the risk of IS in Chinese populations, we carried out the present meta-analysis. No studies have explored the functions of SNP rs918592 to date, so we preliminarily analyzed it using bioinformatics approaches.

## Methods

The present study was conducted following the Preferred Reporting Items for Systematic Reviews and Meta-Analyses (PRISMA) 2020 statement [13]. The PRISMA checklist can be found in Additional file 1.

### Search strategy

We searched the PubMed, Embase, ISI Web of Science, Weipu, China National Knowledge Infrastructure (CNKI), Chinese Biomedical (CBM) and Wanfang databases from inception to November 30, 2023, using the following items: “phosphodiesterase 4D”, “PDE4D”, “rs918592”, “polymorphism”, “stroke”, “cerebral infarction”, “ischemic stroke”, “cerebrovascular disease”, and their synonyms. The references of the identified articles, as well as relevant reviews and meta-analyses, were also manually scanned for other potentially eligible studies.

### Study selection

The selection of studies should be based on the following criteria: (a) case-control, nested case-control, or cohort studies; (b) assessment of the correlation between SNP rs918592 and IS risk in Chinese populations; and (c) using validated techniques to detect SNP rs918592. Articles were excluded if they (a) had no primary findings, (b) were reviews, editorials, case reports, case-only studies, or family-based studies, or (c) were duplicate studies.

### Data extraction

The following information was collected from each qualified study: first author’s name, year of publication, ethnicity of participants, number of cases and controls, mean age of cases and controls, methods for detecting SNP rs918592, matching variables of controls, IS subtypes (if mentioned in the article), as well as number of alleles and genotypes.

Literature screening, data collection, and assessment of study quality were conducted independently by two researchers (X.Y. and G. Z.). The divergences that occurred through the process were settled by discussing with the corresponding author (R. L.).

### Statistical analyses

Hardy-Weinberg equilibrium (HWE) was evaluated by a Chi-square test in the controls. Statistical analyses were performed using STATA 11.0 software (Stata Corporation, College Station, TX). The strength of association between SNP rs918592 and IS risk was calculated using pooled odds ratios (ORs) with 95% confidence intervals (CIs) for two comparisons between different genotypes (AG vs. AA, and GG vs. AA), as well as under dominant (GG + AG vs. AA), additive (G vs. A), and recessive (GG vs. AG + AA) genetic models.

The heterogeneity between studies was assessed with chi-square-based Q-test and *I*^2^ test. When *P* value was above 0.10, a fixed-effects model using the Mantel-Haenszel method was selected for data analysis; otherwise, a random-effects model using the DerSimonian-Laird method was conducted. *I*^2^ metric was used to show the degree of heterogeneity, where 0–25%, 25–50%, 50–75% and 75–100% meant no, moderate, large, and extreme heterogeneity, respectively. The underlying factors causing heterogeneity were explored by meta-regression analysis.

Sensitivity analyses by sequentially removing each study at a time were performed to test the stability of the results. Publication bias was estimated by Begg’s test with funnel plot and Egger’s linear regression test with publication bias plot.

### In Silico bioinformatics analysis

To explore the functions of SNP rs918592, two online prediction websites were used for bioinformatics analysis: HaploReg (http://pubs.broadinstitute.org/mammals/haploreg/haploreg.php) and RegulomeDB (http://regulomedb.org/). HaploReg was applied to discover noncoding genomic annotations for variants and determine their underlying causal correlations with disease pathogenesis. RegulomeDB was utilized for the annotation of variants with regulatory elements by giving ranks. The lower the rank, the more likely it is to have a regulatory function.

## Results

### Eligible studies

**Fig. 1:**
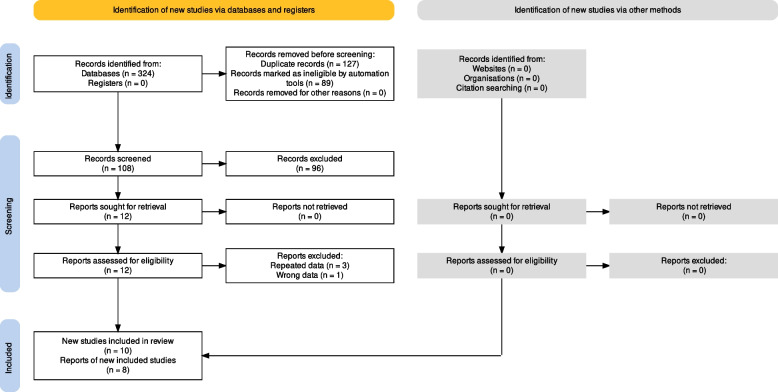
Flow diagram of the literature selection process

After literature search and further screening, 12 articles in total met the inclusive criteria (Fig. 1). Four were subsequently excluded by careful reading of the full text. Xu’s (2008) [14], Bai’s (2011) [15] and Sun’s (2013) [16] studies overlapped with Xu’s (2008) [11], He’s (2012) [12] and Ma’s (2014) [17] studies, respectively, and then were excluded. In Zhang’s (2019) study, SNP87 was incorrectly labeled as rs918592 and actually rs2910829 [18]. Two articles investigated the association in independent populations, so each article was considered as two independent studies [17, 19]. Finally, 10 studies (in 8 articles involving 2,348 IS cases and 2,289 controls) were enrolled in the meta-analysis of the correlation between SNP rs918592 and IS risk (Table 1 and Additional file 2: Table S1) [10–12, 17, 19–22]. Each study design was case-control. The genotypic distribution of one study [22] deviated from HWE expectation in controls (Additional file 2: Table S2).

**Table 1:** Main characteristics of studies included in the meta-analysis of the relationship between SNP rs918592 and the risk of ischemic stroke

First author	Year	Ethnicity	Sample size		Mean age ± SD(year)		Genotyping method	Matching variables of controls	Phenotype
			**Cases**	**Controls**	**Cases**	**Controls**			
Tang JS	2007	Chinese Han	131	112	62.4 ± 9.63	53.2 ± 7.98	PCR–RFLP	Age, gender, smoking, drinking, and BMI	IS
Xu SL	2008	Chinese Han	116	110	65.9 ± 12.4	65.1 ± 12.7	PCR–RFLP	Age and gender	LAA and SVD
He Y	2012	Chinese Han	400	400	61 ± 10	58 ± 10	PCR–RFLP	Age and gender	IS
He Y	2013	Chinese Han	186	232	36.5 ± 6.4	36.8 ± 6.8	PCR–RFLP	Age and gender	early onset IS
Ma JH	2013	Chinese Han	395	395	59.78 ± 11.55		SNaPshot	Age and gender	IS
Ma J	2014	Chinese Han	189	194			PCR–RFLP	Age and gender	IS
Song HJ	2015	Chinese Han	307	227	61.86 ± 8.77	63.35 ± 7.92	SNaPshot	Age, gender, MAP, smoking, HDL, LDL, BMI, and FG	IS with hypertension
Ma JH	2013	Chinese Uyghur	395	395	58.65 ± 10.46		SNaPshot	Age and gender	IS
Ma J	2014	Chinese Uyghur	184	183			PCR–RFLP	Age and gender	IS
Xu MC	2014	Chinese Wa	52	55	59.00 ± 7.75	56.64 ± 8.29	PCR–RFLP and Sanger sequencing	Age, gender, smoking, and diabetes	IS

*PCR–RFLP* polymerase chain reaction-restriction fragment length polymorphism, *BMI* body mass index, *MAP* mean arterial pressure, *HDL* high density lipoprotein, *LDL* low density lipoprotein, *FG* fasting glucose, *IS* ischemic stroke, *LAA* large artery atherosclerosis, *SVD* cerebral small vessel disease

### SNP rs918592 and ischemic Stroke risk

**Fig. 2:**
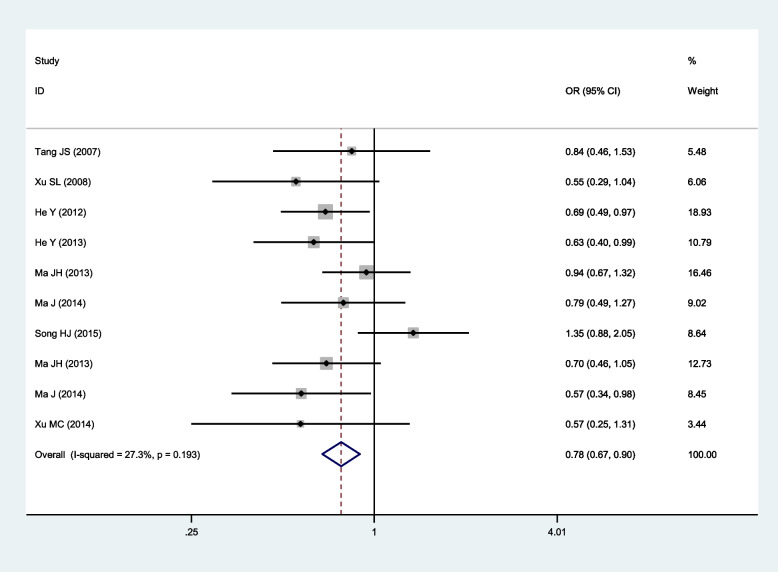
Forest plot for the relationship between the risk of ischemic stroke and SNP rs918592 (AG vs AA) (fixed effects)

**Fig. 3:**
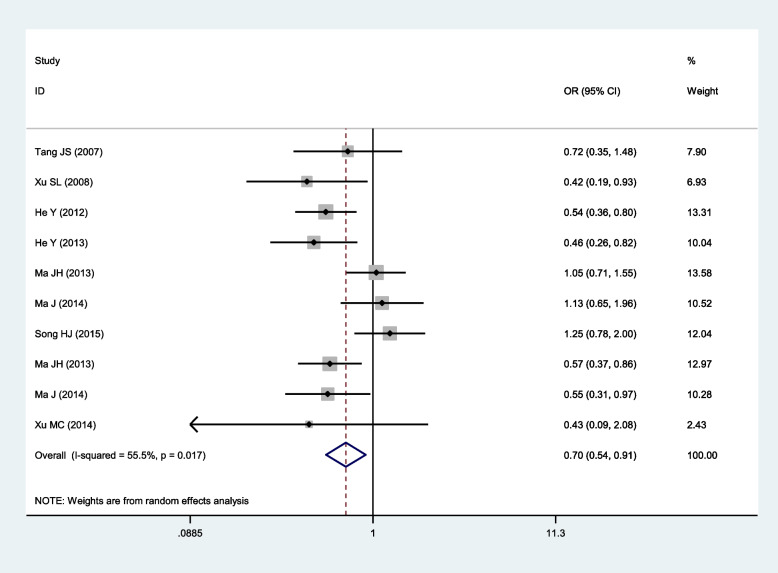
Forest plot for the relationship between the risk of ischemic stroke and SNP rs918592 (GG vs AA) (random effects)

**Fig. 4:**
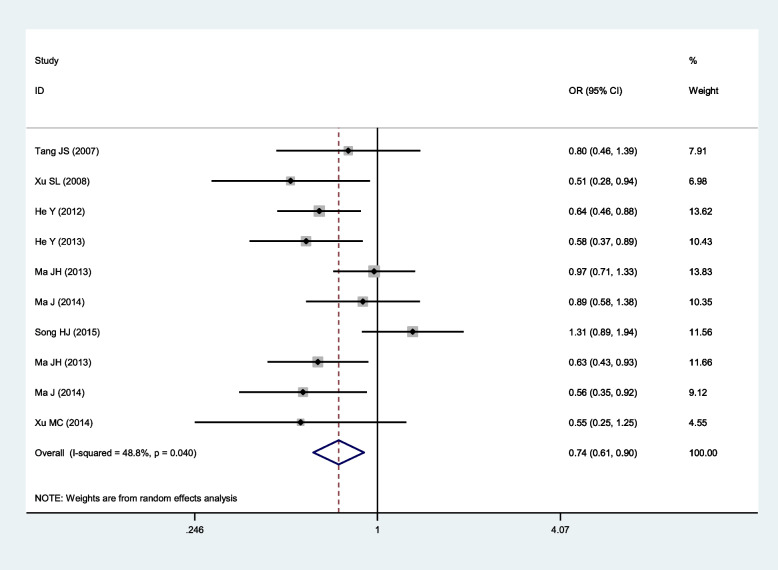
Forest plot for the relationship between the risk of ischemic stroke and SNP rs918592 under the dominant model (AG + GG vs AA) (random effects)

**Fig. 5:**
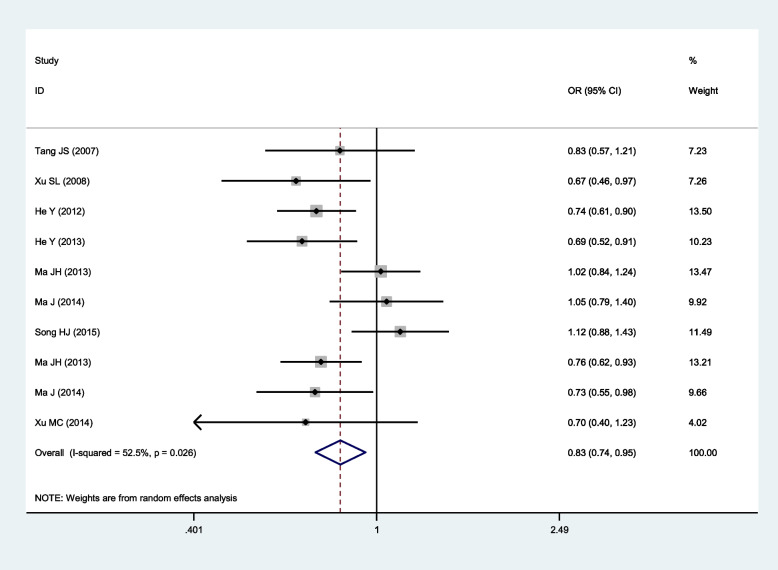
Forest plot for the relationship between the risk of ischemic stroke and SNP rs918592 under the additive model (G vs A) (random effects)

**Fig. 6:**
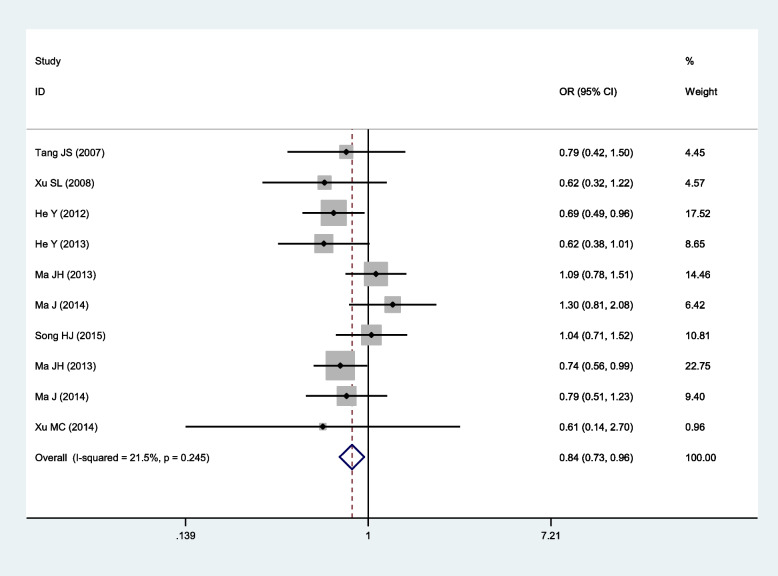
Forest plot for the relationship between the risk of ischemic stroke and SNP rs918592 under the recessive model (GG vs AG + AA) (fixed effects)

Significant association of SNP rs918592 with IS risk was observed in two comparisons and all the three genetic models (Table 2; Figs. 2, 3, 4, 5 and 6). The G allele was related to reduced risk of IS (G vs. A: OR 0.83, 95% CI 0.74–0.95, *P* = 0.005). Among the three genetic models, the dominant model had the smallest OR (GG + AG vs. AA: 0.74, 95% CI 0.61–0.90) and *P* value (0.003), and it might be the best-fitting model. Moderate or large heterogeneity was identified across all studies in the comparison between GG and AA, as well as under the dominant and additive models. Ethnicity, genotyping method, mean age, sample size, and HWE were not the main factors causing heterogeneity, while Song’s (2015) study [21] might be one of the factors causing heterogeneity. After excluding Song’s (2015) study, there was no heterogeneity between studies under the dominant and additive models (Table 2 and Additional file 2: Figs. S1-S5). Notably, all of the patients included in Song’s (2015) study had hypertension, which was markedly different from other studies. All the pooled OR values were not substantially altered after excluding Song’s (2015) study.

**Table 2:** Meta-analysis of the relationship between SNP rs918592 and the risk of ischemic stroke

	**All studies**					**Studies without Song’s (2015) study**				
	**Pooled OR (95% CI)**	***P***_**OR**_	***I*** ^**2**^	***P***_**H**_	**Statistical model**	**Pooled OR (95% CI)**	***P***_**OR**_	***I*** ^**2**^	***P***_**H**_	**Statistical model**
AG vs AA	0.78 (0.67–0.90)	**0.001**	27.3%	0.193	Fixed	0.72 (0.62–0.84)	** < 0.001**	0.0%	0.758	Fixed
GG vs AA	0.70 (0.54–0.91)	**0.008**	55.5%	**0.017**	Random	0.65 (0.51–0.83)	**0.001**	43.1%	**0.080**	Random
Dominant	0.74 (0.61–0.90)	**0.003**	48.8%	**0.040**	Random	0.71 (0.61–0.81)	** < 0.001**	11.9%	0.335	Fixed
Additive	0.83 (0.74–0.95)	**0.005**	52.5%	**0.026**	Random	0.81 (0.74–0.89)	** < 0.001**	37.5%	0.119	Fixed
Recessive	0.84 (0.74–0.96)	**0.010**	21.5%	0.245	Fixed	0.82 (0.71–0.94)	**0.005**	21.3%	0.254	Fixed

*P*_OR_ and *P*_H_ are *P* values for odds ratio and heterogeneity, respectively

*P*_OR_ values significant at *P* < 0.05 and *P*_H_ values significant at *P* < 0.10 are shown in bold

### Sensitivity analyses and publication bias

 When any single study was removed, the pooled OR value was not significantly affected (Additional file 2: Figs. S6-S10), suggesting good stability of the results in the present study. Moreover, the results of Begg’s and Egger’s linear regression tests displayed no significant publication bias (Figs. 7, 8, 9, 10 and 11).

**Fig. 7:**
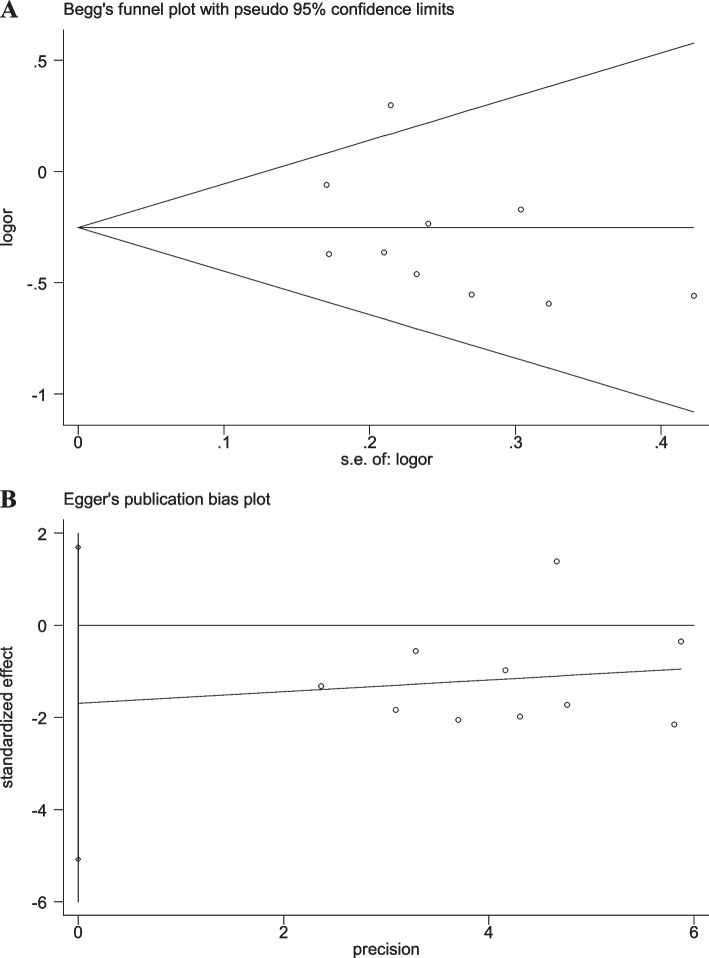
Begg’s funnel and Egger’s publication bias plots for the relationship between the risk of ischemic stroke and SNP rs918592 (AG vs AA) (Begg’s *P* = 0.371, Egger’s *P* = 0.283)

**Fig. 8:**
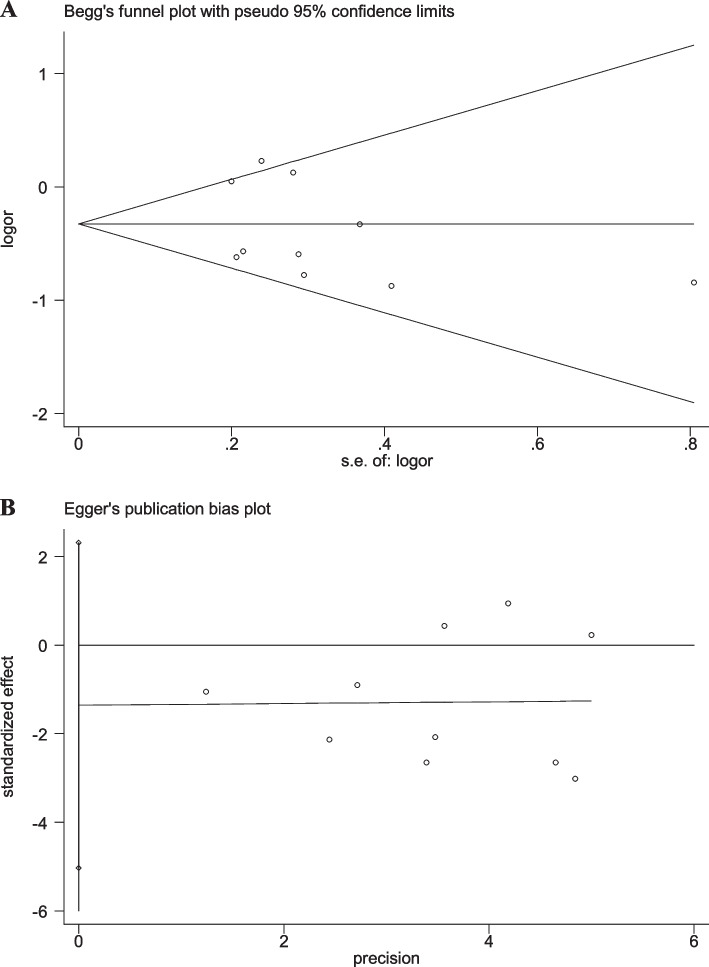
Begg’s funnel and Egger’s publication bias plots for the relationship between the risk of ischemic stroke and SNP rs918592 (GG vs AA) (Begg’s *P* = 0.592, Egger’s *P* = 0.418)

**Fig. 9:**
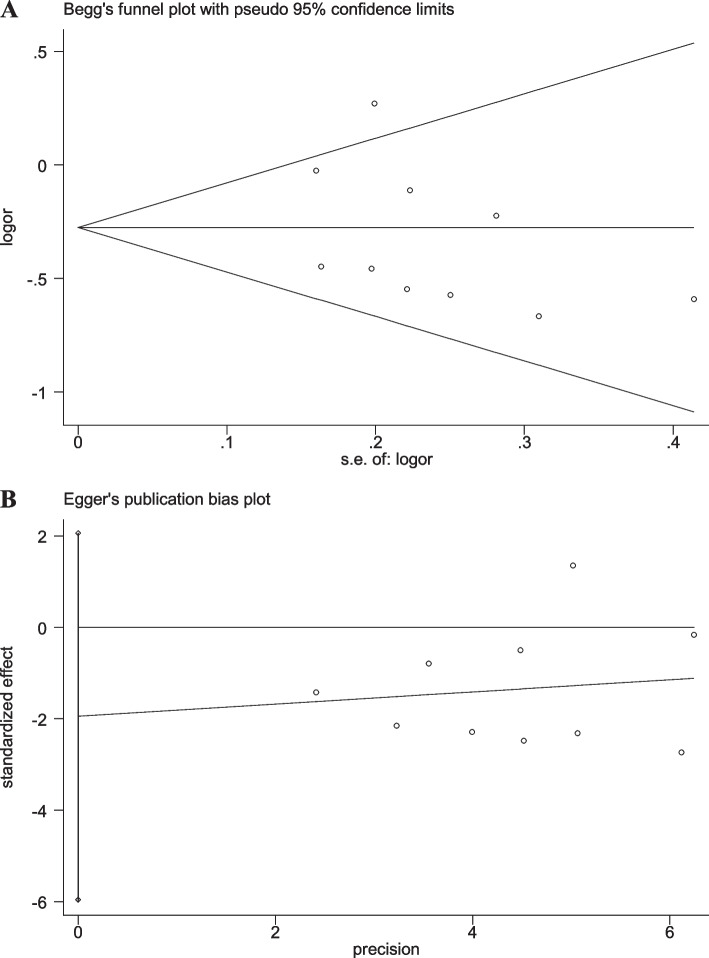
Begg’s funnel and Egger’s publication bias plots for the relationship between the risk of ischemic stroke and SNP rs918592 under the dominant model (AG + GG vs AA) (Begg’s *P* = 0.474, Egger’s *P* = 0.295)

**Fig. 10:**
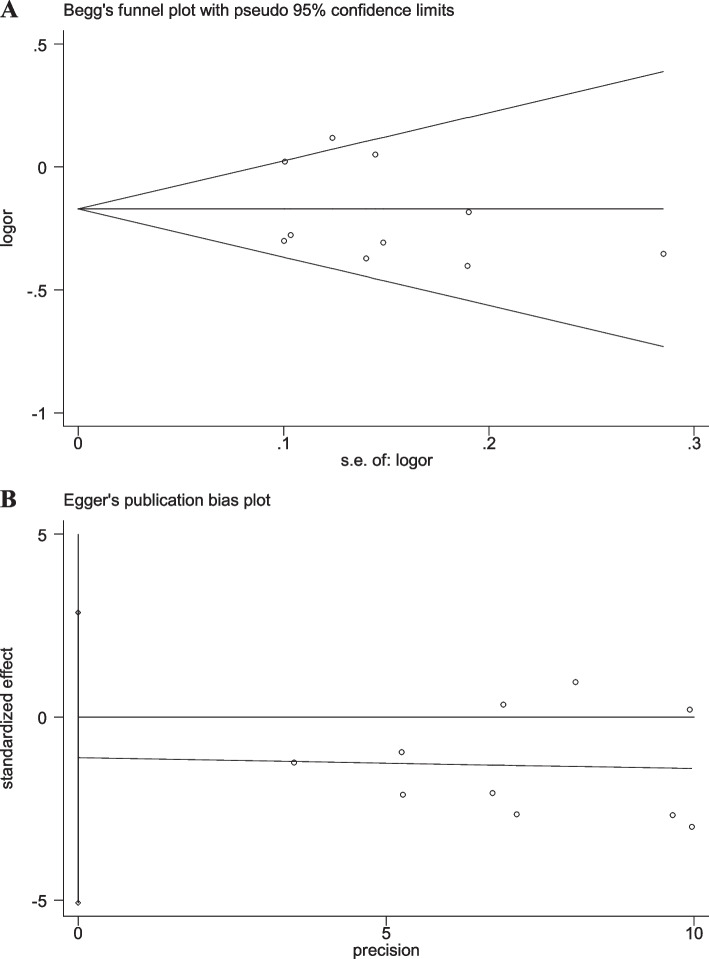
Begg’s funnel and Egger’s publication bias plots for the relationship between the risk of ischemic stroke and SNP rs918592 under the additive model (G vs A) (Begg’s *P* = 1.000, Egger’s *P* = 0.537)

**Fig. 11:**
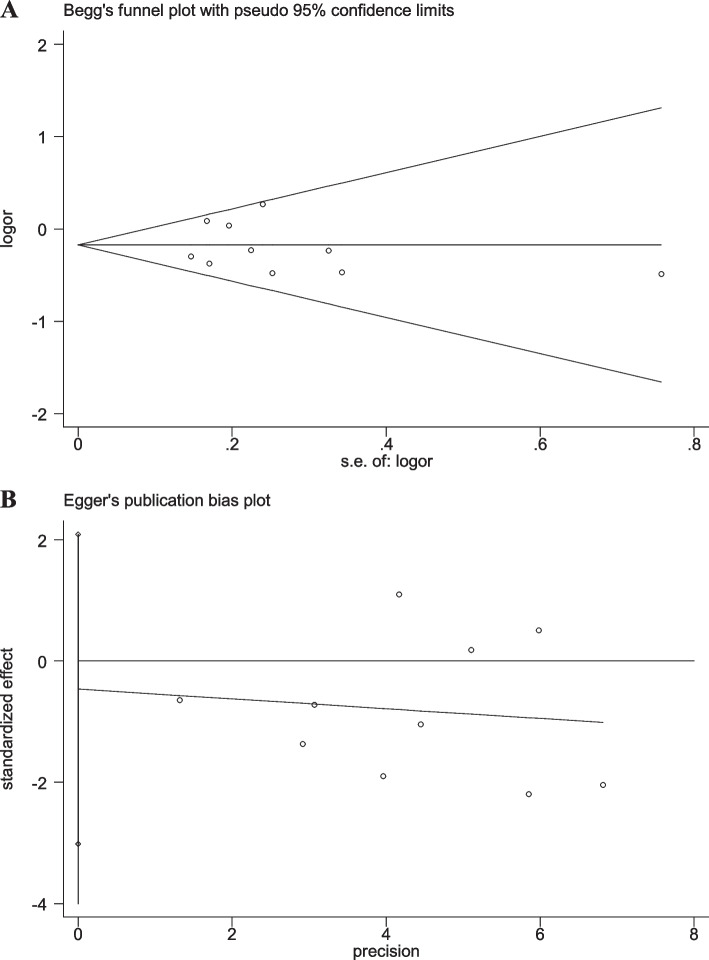
Begg’s funnel and Egger’s publication bias plots for the relationship between the risk of ischemic stroke and SNP rs918592 under the recessive model (GG vs AG + AA) (Begg’s *P* = 1.000, Egger’s *P* = 0.687)

### Functional annotation

We analyzed the functional roles of SNP rs918592 and variants in strong linkage disequilibrium (LD) with it (defined as *r*^2^ ≥ 0.8 with rs918592 in the East Asian (CHB, JPT, and CHS) population) using HaploReg v4.1 (Additional file 2: Table S2). The results showed that SNP rs918592 and the correlated 20 variants mapped to *PDE4D* intronic regions. All of them might affect transcriptional regulatory element activity and be identified as expression quantitative trait loci (eQTL) for prostate androgen-regulated transcript 1 (*PART1*), whose 5’ end overlaps with the 5’ end of *PDE4D* and whose transcript is a long non-coding RNA, in thyroid tissue. Among the correlated variants, two might be located within the histone modification regions of enhancers and one in promoters; two were in DNase I-hypersensitive regions; two had the alteration in transcription factor (TF) binding; one (rs918590) was related to ubiquitin conjugating enzyme E2 E1 (*UBE2E1*) expression in peripheral blood monocytes; and two were located in evolutionarily conserved regions predicted to be functionally constrained according to SiPhy or GERP analysis. As a whole, SNP rs34168777 (*r*^2^ = 0.99) (with evidence of conserved region, enhancer histone mark, DNase I-hypersensitive region, TF-binding, any TF motif, and eQTL hit) might be worthiest of further functional study. The results of RegulomeDB v2.1 also showed that rs34168777 had a rank of 1b (eQTL + TF binding + any motif + DNase Footprint + DNase peak), which was the best ranking among the 21 SNPs (Additional file 2: Table S3). The rank of SNP rs918592 was 1f.

## Discussion

The present meta-analysis revealed that SNP rs918592 was related to the risk of IS in Chinese populations. Although the sample size of the present study was not large, the results were stable in various comparisons and models. So far as we know, this study is the first meta-analysis of the relationship between SNP rs918592 and IS risk in Chinese populations.

In fact, we found only one (Song’s (2006)) study [23] on the correlation between SNP rs918592 and IS risk outside the Chinese populations during literature search. This study focused on early-onset IS in a female population, which showed the A allele of SNP rs918592 was the risk allele in African-Americans and Caucasians, similar to the present study. They also pointed out linkage disequilibrium existed between SNPs rs918592 and rs152312 (SNP 41, related to IS in Icelanders) among Caucasians (LD = 0.66) and African-Americans (LD = 1.0). But the deCODE Genetics group incorrectly labeled SNP41 as rs152312 in 2003 [9], which was corrected to rs12153798 in 2005. That is, actually, rs12153798, rather than rs152312, was associated with IS risk in the Icelandic population. Since both Song’s (2006) study and our meta-analysis suggest that SNP rs918592 is associated with IS risk, we suggest that future studies should be extended not only in the Chinese populations but also in others.

The deCODE Genetics group revealed the association of *PDE4D* variants with IS risk, particularly strong with the risk of cardioembolic (CE) and large artery atherosclerosis (LAA) stroke [9]. We tried to perform the analysis in IS subtypes. There were only two studies for LAA stroke (402 cases and 420 controls) and two studies for small vessel stroke (295 cases and 420 controls) (Additional file 2: Tables S4, S5). Therefore, there were not enough data for meta-analyses of IS subtypes. It is unclear which subtype of IS SNP rs918592 is mainly associated with. It is a shortcoming of this study.

SNP rs918592 is an intron variant of *PDE4D* and may be a causal variant or just a marker in LD with the causal variant. It is necessary to further investigate the functions of SNP rs918592 and variants in LD with it to find the true pathogenic variant. The results of functional prediction showed that SNP rs918592 and its linked 20 variants might have regulatory functions and SNP rs34168777 was the most likely causal variant among them. We only predicted their possible functions, but did not carry out experiments to validate them, which is another shortcoming of this study.

## Conclusion

This study suggests that SNP rs918592 in *PDE4D* may contribute to IS risk in Chinese populations. It provides a better answer for the association of *PDE4D* SNP rs918592 with IS risk in Chinese populations. Larger and more refined studies will be conducive to elucidate this effect on IS, especially on CE and LAA stroke. Further functional studies are also required to identify the causal variant(s).

### Supplementary Information

**Additional file 1.** PRISMA 2020 checklist.

**Additional file 2:** **Table S1. **Allelic distribution of SNP rs918592 in ischemic stroke cases and controls. **Table S2. **Summary of functional annotations for SNP rs918592 and variants in strong LD with rs918592 (defined as *r*^2^≥0.8 with rs918592 in the East Asian population) using HaploReg v4.1. **Table S3. **Summary of functional annotations for SNP rs918592 and variants in strong LD with rs918592 (defined as *r*^2^≥0.8 with rs918592 in the East Asian population) using RegulomeDB v2.1 in GRCh38 assembly. **Table S4. **Main characteristics of studies included in the meta-analysis of the relationship between SNP rs918592 and the risk of ischemic stroke subtypes. **Table S5. **Allelic distribution of SNP rs918592 in the cases and controls of ischemic stroke subtypes. **Figure S1.** Forest plot for the relationship between the risk of ischemic stroke and SNP rs918592 (AG vs AA) (fixed effects) after excluding Song’s (2015) study. **Figure S2.** Forest plot for the relationship between the risk of ischemic stroke and SNP rs918592 (GG vs AA) (random effects) after excluding Song’s (2015) study. **Figure S3. **Forest plot for the relationship between the risk of ischemic stroke and SNP rs918592 under the dominant model (AG+GG vs AA) (fixed effects) after excluding Song’s (2015) study. **Figure S4. **Forest plot for the relationship between the risk of ischemic stroke and SNP rs918592 under the additive model (G vs A) (fixed effects) after excluding Song’s (2015) study. **Figure S5. **Forest plot for the relationship between the risk of ischemic stroke and SNP rs918592 under the recessive model (GG vs AG+AA) (fixed effects) after excluding Song’s (2015) study. **Figure S6. **Sensitivity analysis of the pooled OR coefficients (AG vs AA). CI, confidence interval; OR, odds ratio. **Figure S7. **Sensitivity analysis of the pooled OR coefficients (GG vs AA). CI, confidence interval; OR, odds ratio. **Figure S8. **Sensitivity analysis of the pooled OR coefficients under the dominant model (AG+GG vs AA). CI, confidence interval; OR, odds ratio. **Figure S9. **Sensitivity analysis of the pooled OR coefficients under the additive model (G vs A). CI, confidence interval; OR, odds ratio. **Figure S10. **Sensitivity analysis of the pooled OR coefficients under the recessive model (GG vs AG+AA). CI, confidence interval; OR, odds ratio.

## Data Availability

The datasets supporting the conclusions of this article are included within the article and its Additional file 2: Tables S1-S5 and Figs. S1-S10.

## Abbreviations

IS: Ischemic stroke
PDE4D: Phosphodiesterase 4D
cAMP: cyclic adenosine monophosphate
SNP: Single nucleotide polymorphism
CNKI: China National Knowledge Infrastructure
CBM: Chinese Biomedical
OR: Odds ratio
CI: Confidence interval
LD: Linkage disequilibrium
eQTL: expression quantitative trait loci
PART1: Prostate androgen-regulated transcript 1
TF: Transcription factor
UBE2E1: Ubiquitin conjugating enzyme E2 E1
CE: Cardioembolic
LAA: Large artery atherosclerosis
PCR–RFLP: Polymerase chain reaction-restriction fragment length polymorphism
BMI: Body mass index
MAP: Mean arterial pressure
HDL: High density lipoprotein
LDL: Low density lipoprotein
FG: Fasting glucose
SVD: Cerebral small vessel disease
