# Supplementary material for: PDE4D single nucleotide polymorphism rs918592 is associated with ischemic Stroke risk in Chinese populations: a meta-analysis
Source: BMC Cardiovasc Disord. 2024 Jan 3;24:17. doi: 10.1186/s12872-023-03681-2 (PMC10765709; doi:10.1186/s12872-023-03681-2)
Supplement: Supplementary file 2 — Additional file 2: Table S1. Allelic distribution of SNP rs918592 in ischemic stroke cases and controls. Table S2. Summary of functional annotations for SNP rs918592 and variants in strong LD with rs918592 (defined as r2≥0.8 with rs918592 in the East Asian population) using HaploReg v4.1. Table S3. Summary of functional annotations for SNP rs918592 and variants in strong LD with rs918592 (defined as r2≥0.8 with rs918592 in the East Asian population) using RegulomeDB v2.1 in GRCh38 assembly. Table S4. Main characteristics of studies included in the meta-analysis of the relationship between SNP rs918592 and the risk of ischemic stroke subtypes. Table S5. Allelic distribution of SNP rs918592 in the cases and controls of ischemic stroke subtypes. Figure S1. Forest plot for the relationship between the risk of ischemic stroke and SNP rs918592 (AG vs AA) (fixed effects) after excluding Song’s (2015) study. Figure S2. Forest plot for the relationship between the risk of ischemic stroke and SNP rs918592 (GG vs AA) (random effects) after excluding Song’s (2015) study. Figure S3. Forest plot for the relationship between the risk of ischemic stroke and SNP rs918592 under the dominant model (AG+GG vs AA) (fixed effects) after excluding Song’s (2015) study. Figure S4. Forest plot for the relationship between the risk of ischemic stroke and SNP rs918592 under the additive model (G vs A) (fixed effects) after excluding Song’s (2015) study. Figure S5. Forest plot for the relationship between the risk of ischemic stroke and SNP rs918592 under the recessive model (GG vs AG+AA) (fixed effects) after excluding Song’s (2015) study. Figure S6. Sensitivity analysis of the pooled OR coefficients (AG vs AA). CI, confidence interval; OR, odds ratio. Figure S7. Sensitivity analysis of the pooled OR coefficients (GG vs AA). CI, confidence interval; OR, odds ratio. Figure S8. Sensitivity analysis of the pooled OR coefficients under the dominant model (AG+GG vs AA). CI, confidence interval; OR, [file 12872_2023_3681_MOESM2_ESM.docx]

**Supporting Information to**

***PDE4D* single nucleotide polymorphism rs918592 is associated with ischemic stroke risk in Chinese populations: A meta-analysis**

Xinrui Yu, Guiying Zhang, Xuelei Tang, Rong Lin

| **Table S1. Allelic distribution of SNP rs918592 in ischemic stroke cases and controls.** | | | | | | | | | | |
| --- | --- | --- | --- | --- | --- | --- | --- | --- | --- | --- |
| First author | Year | Ethnicity | Cases | | |  | Controls | | | |
|  |  |  | A(major) | G (minor) | MAF |  | A(major) | G (minor) | MAF | HWE |
| Tang JS | 2007 | Chinese Han | 146 | 102 | 0.411 |  | 267 | 153 | 0.456 | 0.311 |
| Xu SL | 2008 | Chinese Han | 136 | 96 | 0.414 |  | 267 | 153 | 0.514 | 0.125 |
| He Y | 2012 | Chinese Han | 443 | 357 | 0.446 |  | 267 | 153 | 0.521 | 0.255 |
| He Y | 2013 | Chinese Han | 218 | 154 | 0.414 |  | 267 | 153 | 0.506 | 0.236 |
| Ma JH | 2013 | Chinese Han | 406 | 384 | 0.486 |  | 410 | 380 | 0.481 | 0.937 |
| Ma J | 2014 | Chinese Han | 198 | 180 | 0.476 |  | 208 | 180 | 0.464 | 0.943 |
| Song HJ | 2015 | Chinese Han | 293 | 321 | 0.523 |  | 230 | 224 | 0.493 | 0.073 |
| Ma JH | 2013 | Chinese Uyghur | 332 | 458 | 0.580 |  | 280 | 510 | 0.646 | 0.762 |
| Ma J | 2014 | Chinese Uyghur | 183 | 185 | 0.503 |  | 154 | 212 | 0.579 | 0.456 |
| Xu MC | 2014 | Chinese Wa | 70 | 34 | 0.327 |  | 65 | 45 | 0.409 | **0.019** |
| Total |  |  | 2348 | |  |  | 2289 | |  |  |
| Abbreviations: MAF, minor allele frequency; HWE, Hardy-Weinberg equilibrium.  *P* values significant at *P*<0.05 are shown in bold. | | | | | | | | | | |

| **Table S2. Summary of functional annotations for SNP rs918592 and variants in strong LD with rs918592 (defined as *r*^2^≥0.8 with rs918592 in the East Asian population) using HaploReg v4.1.** | | | | | | | | | | | | | | | | | | | | | | |
| --- | --- | --- | --- | --- | --- | --- | --- | --- | --- | --- | --- | --- | --- | --- | --- | --- | --- | --- | --- | --- | --- | --- |
|  | **Position (hg38)** | **LD** | | **Variant** | **Ref** | **Alt** | **Frequency of alternate allele** | | | | **GERP cons** | **SiPhy cons** | **Promoter histone marks** | **Enhancer histone marks** | **DNase** | **Proteins bound** | **Motifs changed** | **NHGRI/EBI GWAS hits** | **GRASP QTL hits** | **Selected eQTL**  **hits** | **GENCODE genes** | **dbSNP func annot** |
|  |  | ***r*²** | **D'** |  |  |  | **AFR** | **AMR** | **ASN** | **EUR** |  |  |  |  |  |  |  |  |  |  |  |  |
| 1 | 60374208 | 0.88 | 0.95 | rs1363862 | G | A | 0.74 | 0.36 | 0.52 | 0.25 |  |  | ESDR |  |  |  | 4 altered motifs |  |  | 1 hit | PDE4D | intronic |
| 2 | 60375352 | 0.83 | 0.92 | rs1477352 | A | C | 0.36 | 0.23 | 0.51 | 0.13 |  |  |  |  |  |  | p53 |  |  | 1 hit | PDE4D | intronic |
| 3 | 60378365 | 0.89 | 0.95 | rs6860887 | C | T | 0.73 | 0.37 | 0.52 | 0.26 |  |  |  | FAT |  |  | AIRE,HNF4 |  |  | 1 hit | PDE4D | intronic |
| 4 | 60382015 | 0.90 | 0.95 | rs1115372 | G | A | 0.73 | 0.37 | 0.52 | 0.26 |  |  |  |  |  |  | Fox,Hmbox1 |  |  | 1 hit | PDE4D | intronic |
| 5 | 60389746 | 0.99 | 1.00 | rs12189147 | T | C | 0.73 | 0.35 | 0.52 | 0.24 |  |  |  |  |  |  | NF-I |  |  | 1 hit | PDE4D | intronic |
| 6 | 60395976 | 0.99 | 1.00 | rs17839379 | T | C | 0.73 | 0.35 | 0.52 | 0.24 |  |  |  |  | 4 tissues | CTCF,RAD21 | 4 altered motifs |  |  | 1 hit | PDE4D | intronic |
| 7 | 60401476 | 1.00 | 1.00 | **rs918592** | C | T | 0.71 | 0.30 | 0.52 | 0.21 |  |  |  |  |  |  | 5 altered motifs |  |  | 1 hit | PDE4D | intronic |
| 8 | 60401660 | 0.99 | 1.00 | rs918591 | G | A | 0.73 | 0.33 | 0.52 | 0.24 |  | conserved |  |  |  |  | Irx,Myc |  |  | 1 hit | PDE4D | intronic |
| 9 | 60401981 | 1.00 | 1.00 | rs918590 | A | C | 0.53 | 0.29 | 0.52 | 0.21 |  |  |  |  |  |  | 4 altered motifs |  | 1 hit | 1 hit | PDE4D | intronic |
| 10 | 60402847 | 0.99 | 1.00 | rs2910629 | C | G | 0.73 | 0.34 | 0.52 | 0.24 |  |  |  |  |  |  | AFP1 |  |  | 1 hit | PDE4D | intronic |
| 11 | 60403384 | 0.99 | 1.00 | [rs2042315](https://pubs.broadinstitute.org/mammals/haploreg/detail_v4.1.php?query=&id=rs2042315) | T | C | 0.73 | 0.35 | 0.52 | 0.24 |  |  |  |  |  |  | EBF,GATA,STAT |  |  | 1 hit | PDE4D | intronic |
| 12 | 60404346 | 0.99 | 1.00 | rs34168777 | CT | C | 0.73 | 0.33 | 0.52 | 0.24 | conserved |  |  | STRM, BRN, GI | 6 tissues | CEBPB | 6 altered motifs |  |  | 1 hit | PDE4D | intronic |
| 13 | 60406111 | 0.98 | 0.99 | rs1423247 | C | T | 0.73 | 0.33 | 0.52 | 0.24 |  |  |  |  |  |  | 11 altered motifs |  |  | 1 hit | PDE4D | intronic |
| 14 | 60409172 | 0.92 | 0.98 | rs13159422 | C | T | 0.61 | 0.31 | 0.51 | 0.23 |  |  |  |  |  |  | CEBPB |  |  | 1 hit | PDE4D | intronic |
| 15 | 60411199 | 0.87 | 0.96 | rs789389 | T | C | 0.62 | 0.33 | 0.53 | 0.24 |  |  |  |  |  |  | GATA,HP1-site-factor,Pou2f2 |  |  | 1 hit | PDE4D | intronic |
| 16 | 60421873 | 0.85 | 0.94 | rs42968 | C | T | 0.58 | 0.35 | 0.52 | 0.31 |  |  |  |  |  |  | Myc,YY1 |  |  | 1 hit | PDE4D | intronic |
| 17 | 60423252 | 0.81 | 0.91 | rs37700 | G | C | 0.58 | 0.35 | 0.51 | 0.31 |  |  |  |  |  |  | Alx4 |  |  | 1 hit | PDE4D | intronic |
| 18 | 60424649 | 0.85 | 0.94 | rs37695 | C | T | 0.62 | 0.36 | 0.52 | 0.31 |  |  |  |  |  |  | Zic |  |  | 1 hit | PDE4D | intronic |
| 19 | 60425979 | 0.82 | 0.91 | rs37693 | G | A | 0.60 | 0.35 | 0.52 | 0.30 |  |  |  |  |  |  | 6 altered motifs |  |  | 1 hit | PDE4D | intronic |
| 20 | 60427258 | 0.87 | 0.96 | rs37691 | G | A | 0.62 | 0.36 | 0.53 | 0.31 |  |  |  |  |  |  | 6 altered motifs |  |  | 1 hit | PDE4D | intronic |
| 21 | 60435814 | 0.84 | 0.93 | rs37685 | C | T | 0.62 | 0.36 | 0.52 | 0.31 |  |  |  |  |  |  | 4 altered motifs |  |  | 1 hit | PDE4D | intronic |
| SNP rs918592 is labeled in bold.  Abbreviations: LD, linkage disequilibrium; Ref, reference; Alt, alternate; AFR, African (YRI, LWK, ASW); AMR, American (MXL, CLM, PUR); ASN, East Asian (CHB, JPT, CHS); EUR, European (CEU, TSI, GBR, FIN, IBS); GERP cons, sequence constraint by GERP; SiPhy cons, sequence constraint by SiPhy; DNase, deoxyribonuclease; GWAS, genome-wide association study; QTL, quantitative trait locus; eQTL, expression quantitative trait locus; func annot, functional annotation. | | | | | | | | | | | | | | | | | | | | | | |

| **Table S3. Summary of functional annotations for SNP rs918592 and variants in strong LD with rs918592 (defined as *r*^2^≥0.8 with rs918592 in the East Asian population) using RegulomeDB v2.1 in GRCh38 assembly.** | | | | | | | | | | | | |
| --- | --- | --- | --- | --- | --- | --- | --- | --- | --- | --- | --- | --- |
|  | **dbSNP IDs** | **Rank** | **Score** | **ChIP** | **DNase** | **Footprint** | **Footprint_matched** | **IC_matched_max** | **IC_max** | **PWM** | **PWM_matched** | **QTL** |
| 1 | rs1363862 | 7 | 0.51392 | FALSE | FALSE | FALSE | FALSE | 0 | 0 | FALSE | FALSE | **TRUE** |
| 2 | rs1477352 | 7 | 0.51392 | FALSE | FALSE | FALSE | FALSE | 0 | 0 | FALSE | FALSE | **TRUE** |
| 3 | rs6860887 | 7 | 0.51392 | FALSE | FALSE | FALSE | FALSE | 0 | 0 | FALSE | FALSE | **TRUE** |
| 4 | rs1115372 | 7 | 0.51392 | FALSE | FALSE | FALSE | FALSE | 0 | 0 | FALSE | FALSE | **TRUE** |
| 5 | rs12189147 | 7 | 0.51392 | FALSE | FALSE | FALSE | FALSE | 0 | 0 | FALSE | FALSE | **TRUE** |
| 6 | rs17839379 | 1f | 0.55436 | **TRUE** | **TRUE** | FALSE | FALSE | 0 | 0 | FALSE | FALSE | **TRUE** |
| 7 | **rs918592** | 1f | 0.55324 | **TRUE** | FALSE | FALSE | FALSE | 0 | 0 | FALSE | FALSE | **TRUE** |
| 8 | rs918591 | 1f | 0.55436 | **TRUE** | **TRUE** | FALSE | FALSE | 0 | 0 | FALSE | FALSE | **TRUE** |
| 9 | rs918590 | 1f | 0.55324 | **TRUE** | FALSE | FALSE | FALSE | 0 | 0 | FALSE | FALSE | **TRUE** |
| 10 | rs2910629 | 7 | 0.51392 | FALSE | FALSE | FALSE | FALSE | 0 | 0 | FALSE | FALSE | **TRUE** |
| 11 | rs2042315 | 1f | 0.55324 | **TRUE** | FALSE | FALSE | FALSE | 0 | 0 | FALSE | FALSE | **TRUE** |
| 12 | rs34168777 | 1b | 0.94075 | **TRUE** | **TRUE** | **TRUE** | FALSE | 0 | 0 | **TRUE** | FALSE | **TRUE** |
| 13 | rs1423247 | 7 | 0.51392 | FALSE | FALSE | FALSE | FALSE | 0 | 0 | FALSE | FALSE | **TRUE** |
| 14 | rs13159422 | 1f | 0.55324 | **TRUE** | FALSE | FALSE | FALSE | 0 | 0 | FALSE | FALSE | **TRUE** |
| 15 | rs789389 | 7 | 0.51392 | FALSE | FALSE | FALSE | FALSE | 0 | 0 | FALSE | FALSE | **TRUE** |
| 16 | rs42968 | 7 | 0.51392 | FALSE | FALSE | FALSE | FALSE | 0 | 0 | FALSE | FALSE | **TRUE** |
| 17 | rs37700 | 7 | 0.51392 | FALSE | FALSE | FALSE | FALSE | 0 | 0 | FALSE | FALSE | **TRUE** |
| 18 | rs37695 | 7 | 0.51392 | FALSE | FALSE | FALSE | FALSE | 0 | 0 | FALSE | FALSE | **TRUE** |
| 19 | rs37693 | 6 | 0.1625 | FALSE | FALSE | FALSE | FALSE | 0 | 0.46 | **TRUE** | FALSE | **TRUE** |
| 20 | rs37691 | 1f | 0.55436 | **TRUE** | **TRUE** | FALSE | FALSE | 0 | 0 | FALSE | FALSE | **TRUE** |
| 21 | rs37685 | 1f | 0.22271 | FALSE | **TRUE** | FALSE | FALSE | 0 | 0 | FALSE | FALSE | **TRUE** |
| SNP rs918592 is labeled in bold.  Abbreviations: LD, linkage disequilibrium; ChIP, Chromatin immunoprecipitation; DNase, deoxyribonuclease; IC_matched_max, maximum information content change of matched TF motif; IC_max, maximum information content change of TF motif; PWM, position weight matrix; QTL, quantitative trait locus.  Reference:  Dong S, Boyle AP. Predicting functional variants in enhancer and promoter elements using RegulomeDB. *Hum Mutat*. 2019; 40(9): 1292-1298. doi:10.1002/humu.23791.  Dong S, Zhao N, Spragins E, Kagda MS, Li M, Assis P, Jolanki O, Luo Y, Cherry JM, Boyle AP, Hitz1 BC. Annotating and prioritizing human non-coding variants with RegulomeDB. *bioRxiv*. 2022. doi:10.1101/2022.10.18.512627. | | | | | | | | | | | | |

| **Table S4. Main characteristics of studies included in the meta-analysis of the relationship between SNP rs918592 and the risk of ischemic stroke subtypes.** | | | | | | | | |
| --- | --- | --- | --- | --- | --- | --- | --- | --- |
| First author | Year | Ethnicity | Sample size | | Mean age±SD(year) | | Genotyping method | Matching variables of controls |
|  |  |  | Cases | Controls | Cases | Controls |  |  |
| **Large artery atherosclerotic stroke** | | | | | | | | |
| Xu SL | 2008 | Chinese Han | 87 | 110 | 65.3±13.2 | 65.1±12.7 | PCR-RFLP | Age and gender |
| Ma JH | 2013 | Chinese Han and Uyghur | 315 | 790 |  | 59.28±10.76 | SNaPshot | Age and gender |
| **Cerebral small vessel disease** | | | | | | | | |
| Xu SL | 2008 | Chinese Han | 29 | 110 | 67.9±9.6 | 65.1±12.7 | PCR-RFLP | Age and gender |
| Ma JH | 2013 | Chinese Han and Uyghur | 266 | 790 |  | 59.28±10.76 | SNaPshot | Age and gender |
| Abbreviations: PCR-RFLP, polymerase chain reaction-restriction fragment length polymorphism. | | | | | | | | |

| **Table S5. Allelic distribution of SNP rs918592 in the cases and controls of ischemic stroke subtypes.** | | | | | | | | | | |
| --- | --- | --- | --- | --- | --- | --- | --- | --- | --- | --- |
| First author | Year | Ethnicity | Cases | | |  | Controls | | | |
|  |  |  | A | G | MAF |  | A | G | MAF | HWE |
| **Large artery atherosclerotic stroke** | | | | | | | | | | |
| Xu SL | 2008 | Chinese Han | 99 | 75 | 0.431 |  | 267 | 153 | 0.514 | 0.125 |
| Ma JH | 2013 | Chinese Han and Uyghur | 315 | 315 | 0.500 |  | 267 | 153 | 0.563 | 0.360 |
| Total |  |  | 402 | |  |  | 420 | |  |  |
| **Cerebral small vessel disease** | | | | | | | | | | |
| Xu SL | 2008 | Chinese Han | 37 | 21 | 0.362 |  | 267 | 153 | 0.514 | 0.125 |
| Ma JH | 2013 | Chinese Han and Uyghur | 229 | 303 | 0.570 |  | 267 | 153 | 0.563 | 0.360 |
| Total |  |  | 295 | |  |  | 420 | |  |  |
| Abbreviations: MAF, minor allele frequency; HWE, Hardy-Weinberg equilibrium. | | | | | | | | | | |

**Figure S1.** Forest plot for the relationship between the risk of ischemic stroke and SNP rs918592 (AG vs AA) (fixed effects) after excluding Song’s (2015) study.

**Figure S2.** Forest plot for the relationship between the risk of ischemic stroke and SNP rs918592 (GG vs AA) (random effects) after excluding Song’s (2015) study.

**Figure S3.** Forest plot for the relationship between the risk of ischemic stroke and SNP rs918592 under the dominant model (AG+GG vs AA) (fixed effects) after excluding Song’s (2015) study.

**Figure S4.** Forest plot for the relationship between the risk of ischemic stroke and SNP rs918592 under the additive model (G vs A) (fixed effects) after excluding Song’s (2015) study.

**Figure S5.** Forest plot for the relationship between the risk of ischemic stroke and SNP rs918592 under the recessive model (GG vs AG+AA) (fixed effects) after excluding Song’s (2015) study.

**Figure S6.** Sensitivity analysis of the pooled OR coefficients (AG vs AA). CI, confidence interval; OR, odds ratio.

**Figure S7.** Sensitivity analysis of the pooled OR coefficients (GG vs AA). CI, confidence interval; OR, odds ratio.

**Figure S8.** Sensitivity analysis of the pooled OR coefficients under the dominant model (AG+GG vs AA). CI, confidence interval; OR, odds ratio.

**Figure S9.** Sensitivity analysis of the pooled OR coefficients under the additive model (G vs A). CI, confidence interval; OR, odds ratio.

**Figure S10.** Sensitivity analysis of the pooled OR coefficients under the recessive model (GG vs AG+AA). CI, confidence interval; OR, odds ratio.
